# Statistical analysis of bank deposits dataset

**DOI:** 10.1016/j.dib.2018.03.096

**Published:** 2018-03-26

**Authors:** Pelumi E. Oguntunde, Hilary I. Okagbue, Patience I. Adamu, Omoleye A. Oguntunde, Sola J. Oluwatunde, Abiodun A. Opanuga

**Affiliations:** a Department of Mathematics, Covenant University, Ota, Nigeria; b Department of Economics and Development Studies, Covenant University, Ota, Nigeria; c Department of Computer Science, Caleb University, Lagos State, Nigeria

**Keywords:** Account, Bank, Deposit, Security, Turkey pairwise comparison, Statistics

## Abstract

This article presents the statistical analysis of the deposit activities in each of the account types of a leading bank in Nigeria. The mean effect of these account types on the bank was determined using analysis of variance (ANOVA). Further test which include the Tukey's simultaneous test for differences of means was also conducted.

**Specifications Table**

**Table:** 

Subject area	Economics
More specific subject area	Banking and Finance, Social Statistics
Type of data	Table and text file
How data was acquired	Secondary data
Data format	Raw and partially analyzed (Descriptive and Inferential)
Experimental factors	Data sets on the amount of money deposited in a bank in different account types
Experimental features	Observations on the number of customers that made deposit into the six various accounts of the bank and the amount they deposited.
Data source location	The data was obtained from one of the leading banks in Nigeria
Data accessibility	All the data are available this data article

**Value of the data**

• The data is useful in calculating loan to deposit ratio.
• The data could be used as one of vital tools in assessing bank competitiveness [1].
• The data analysis could be helpful in detecting non-performing loans (NPL) in credit management [2].
• The data could be helpful in monitoring off balance sheet engagements [3].
• The data could be used to monitor compliance to banking decision making and strategy implementation; for example, innovative savings products [4], [5], [6].
• The data analysis can be applied to monitor statutory policies and regulation; for example, the effect of monetary policies [7].
• The data can be extended to include behavioral attitudes and customer preferences for some types of accounts.

## 1 Data

The data in this article involves the amount of money (in Naira) deposited into six different account types available in a leading bank in Nigeria on a particular day in year 2017. It also gives information on the number of people that make deposits into the various account types.

The bank used has six different account types which we denote as Account Type 1 (Savings), Account Type 2 (Current), Account Type 3 (Corporate), Account Type 4, Account Type 5 and Account Type 6. Since the data is sensitive and a real life data, we would like to protect the privacy policy of the bank. Descriptive statistics was used to summarize the data and to provide plots for proper visualization and understanding. SPSS version 20 and Minitab version 17 were used for the analyses in this paper.

The data set is summarized in Table 1.

**Table 1:** Summary statistics of the dataset.

Account Types	1	2	3	4	5	6
No. of Depositors	30	15	6	8	7	4
Minimum (#)	3000	130,000	180,000	12,000	8000	15,000
Maximum (#)	130,000	850,000	700,000	70,000	80,000	80,000
Sum (#)	649,000	6,663,000	2,192,000	249,000	256,000	132,000
Mean (#)	21,633.33	444,200.00	365,333.33	31,125.00	36,671.43	33,000

The information contained in Table 1 shows that more people patronize account type 1 which is savings account than any other account types but the total money deposited in the account is not necessarily the largest. The account type that attracts the highest deposits is account type 2 (current account), though, the number of depositors for this account type is not the highest but on the average, customers deposited the highest amount of money there. This is reasonable because in the real sense, current account holders could either be for personal, businesses, and corporate organizations.

**Fig. 1:**
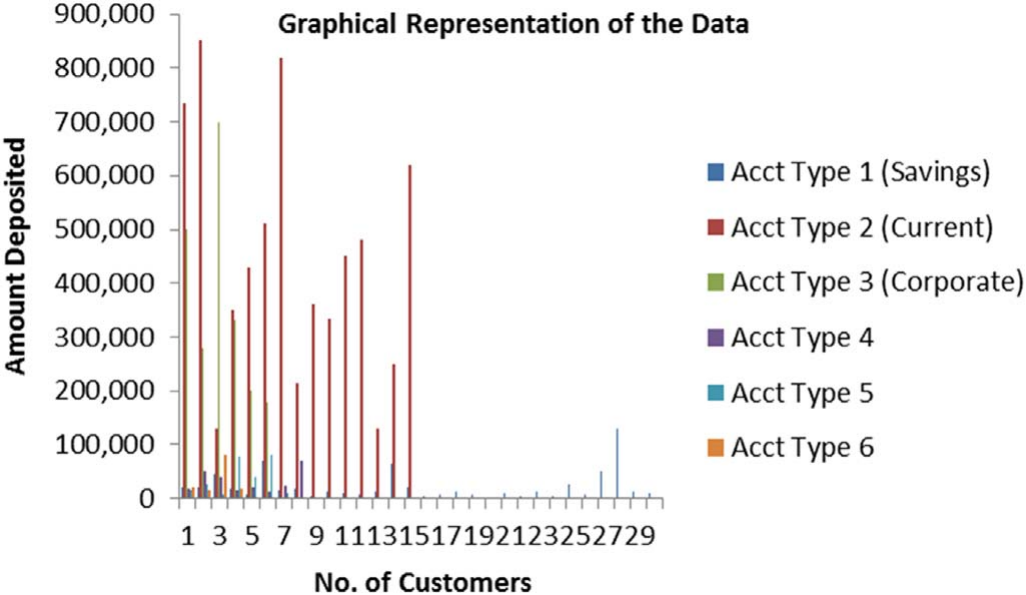
The chart representing the amount of deposits and account types.

A chart that summarizes the whole dataset is presented in Fig. 1.

**Fig. 2:**
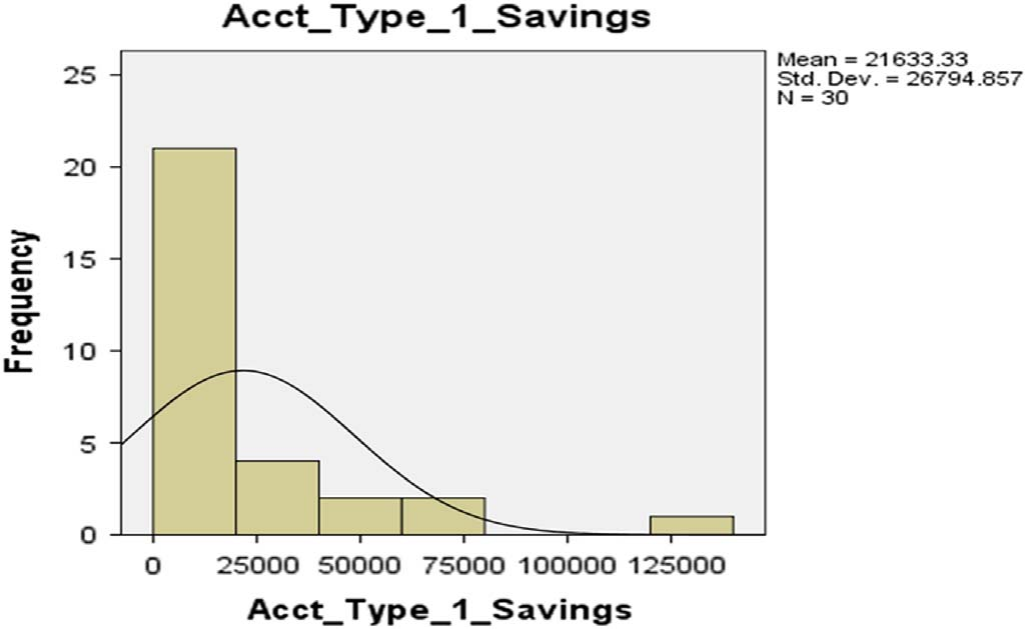
The histogram for Savings Account (Account Type 1).

**Fig. 3:**
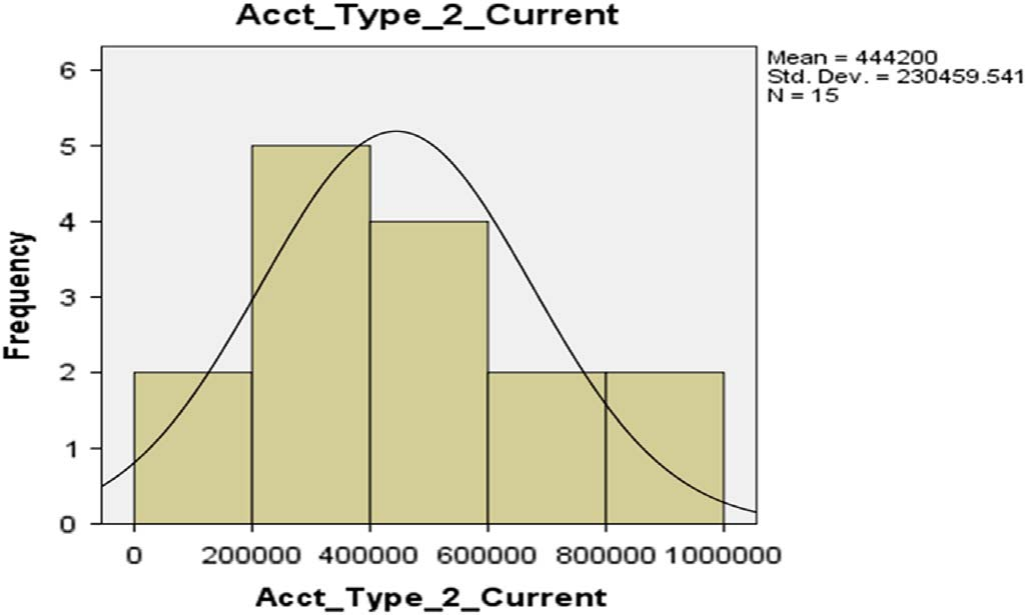
The histogram for Current Account (Account Type 2).

**Fig. 4:**
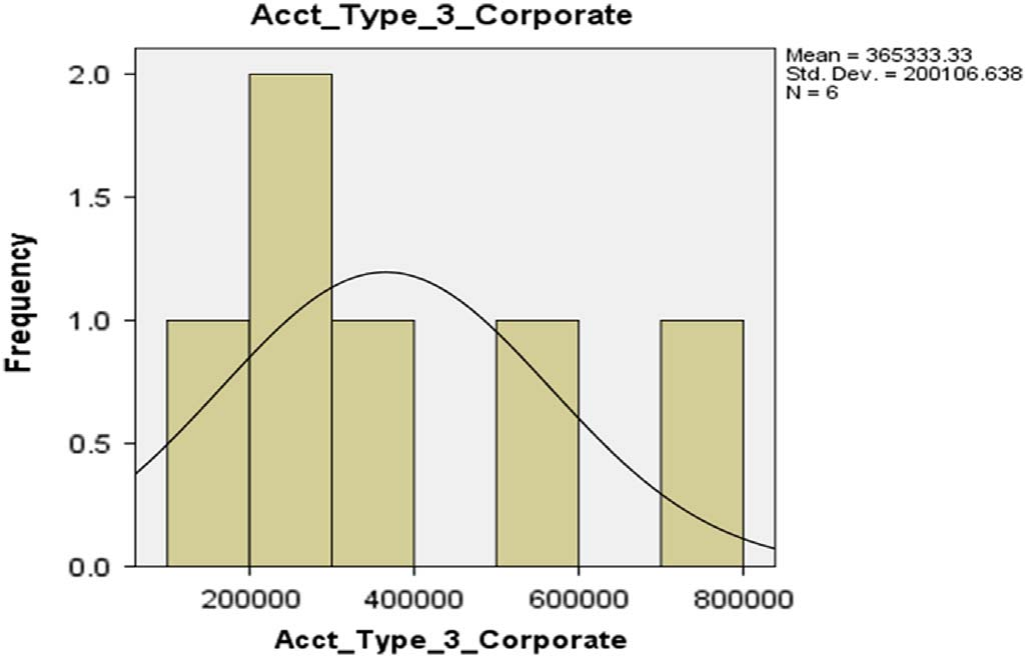
The histogram for Corporate Account (Account Type 3).

**Fig. 5:**
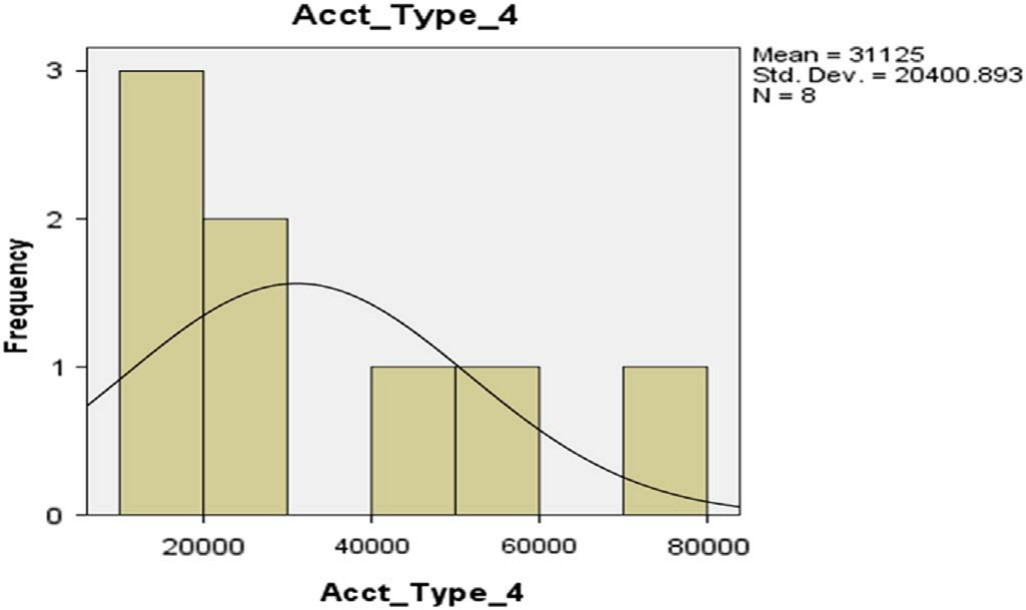
The histogram for Account Type 4.

**Fig. 6:**
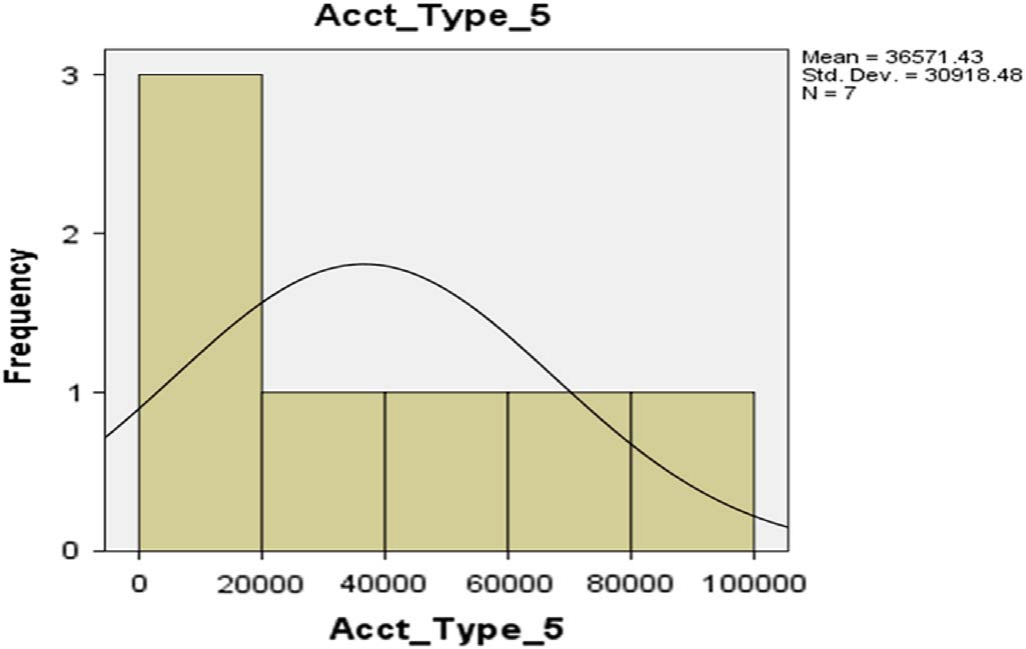
The histogram for Account Type 5.

**Fig. 7:**
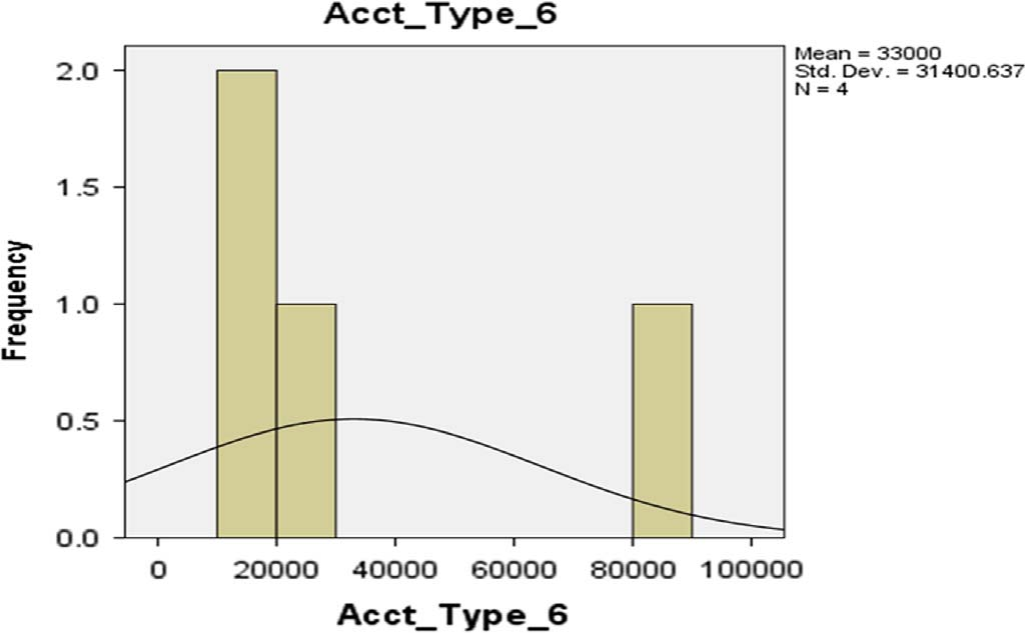
The histogram for Account Type 6.

The deposit patterns for account types 1–6 are provided in form of histogram in Fig. 2, Fig. 3, Fig. 4, Fig. 5, Fig. 6, Fig. 7 respectively.

**Fig. 8:**
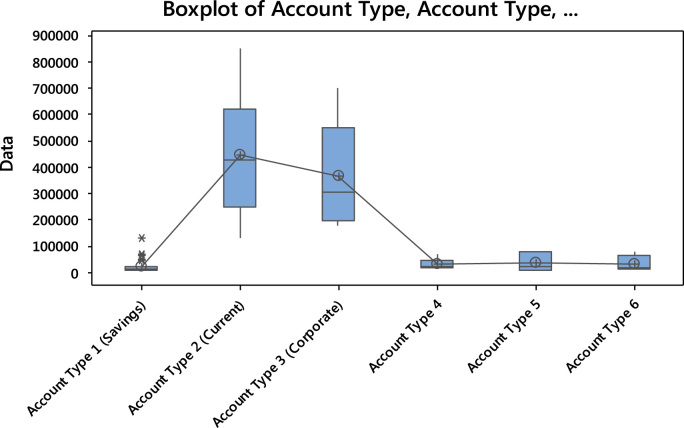
A Boxplot representing the data set.

Also, the boxplot representing the mean amount deposited in the various account types is displayed in Fig. 8.

The impact of the current account is also being identified in the plot provided in Fig. 8.

The mean deposit in each account type with their respective 95% Confidence Interval (C.I) is displayed in Table 2.

**Table 2:** 95% confidence interval for the mean.

Account Type	*N*	Mean	Standard deviation	95% C. I
Account Type 1 (Savings)	30	21633	26795	(−23413, 66679)
Account Type 2 (Current)	15	444200	230460	(380495, 507905)
Account Type 3 (Corporate)	6	365333	200107	(264607, 466059)
Account Type 4	8	31125	20401	(−56106, 118356)
Account Type 5	7	36571	30918	(−56683, 129826)
Account Type 6	4	33000	31401	(−90364, 156364)

**Fig. 9:**
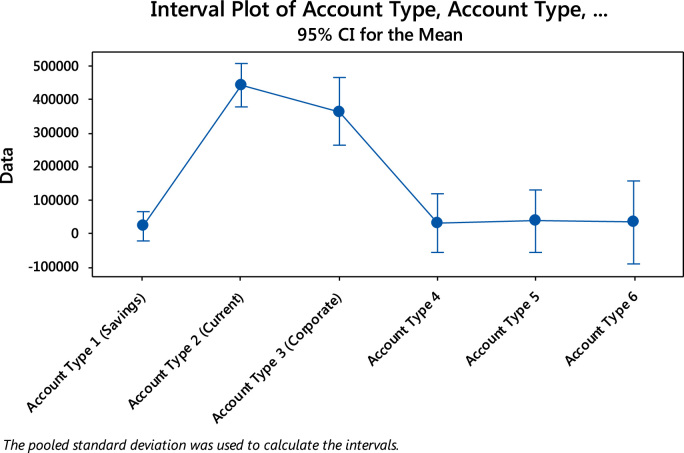
A plot for the 95% C.I for the mean amount of deposits.

The 95% confidence interval plot for the mean of the amount deposited in the various account types is displayed in Fig. 9.

## 2 Experimental design, materials and methods

Analysis of variance has traditionally been used to investigate mean effects of groups of subjects. In this research, a one-way ANOVA is applied. ANOVA and other statistical tools have been applied to the analysis of economic data such as in econometric models, credit management, accounting and audit and many others which are too numerous to enumerate. Furthermore, statistical tools are often combined with other tools for better analysis. Some examples include: macroeconomic volatility generation [8], economic impact of transportation [9], economic impact of professional negotiation [10], Gross Domestic product and exchange rate [11], economic impact of tourism [12], income inequality [13], the effects of expenditure [14], human capital in energy growth [15], quality of life [16], economic impact of portfolio selection [17], economics of refugees and asylum seekers [18], economic recovery [19] and energy needs for economic development [20].

Since we are dealing with a one-way ANOVA, the underlying model is:

$$Y_{ij}=\mu \;+\;\alpha_{ij}\;+\;e_{ij}$$

where $Y_{ij}$ is the *j*th observation in the *i*th treatment, μ is the overall mean, $\alpha_{ij}$ is the effect of treatment *i*, $e_{ij}$ is the error term

The specific hypothesis used is:

H_0_: The mean deposits in all the account types are equal Versus
H_1_: The mean deposits are not equal for at least one of the account types

However, Minitab version 17 was used for the analysis of variance (ANOVA) and further tests. Also, the level of significance used for all the analyses is 0.05. The result is displayed in Table 3.

**Table 3:** Analysis of variance (ANOVA) table.

Source of variation (SV)	Degree of freedom (df)	Sum of square (SS)	Mean square (MS)	*F*	*P*-value
Account types	5	2.32688E+12	4.65377E+11	30.51	0.000
Error	64	9.76204E+11	15,253,184,208		
Total	69	3.30309E+12			

**Decision Rule**: Reject H_0_ if *p*-value is less or equal to the level of significance.

**Decision**: We reject H_0_ since *p*-value (0.000) is less than the level of significance (0.05).

**Inference**: The mean deposits are not equal for at least one of the account types.

The ANOVA model is summarized in Table 4.

**Table 4:** Model summary.

Statistic	Value
Pooled standard deviation	123,504
R-square	70.45%
R-square (Adjusted)	68.14%
R-square (predicted)	64.24%

### 2.1 Turkey pairwise comparisons

Since H_0_ was rejected, we are interested in knowing which pair of the means is actually significantly different from each other using Turkey pairwise comparisons. The means are paired, the differences between the means are calculated and the Tukey's simultaneous test for differences of means of the deposits is obtained. The result is displayed in Table 5.

**Table 5:** Tukey simultaneous tests for differences of means.

Difference between means	Difference	Standard error	95% C.I	*T*-value	*p*-value
Acct Type 2 - Acct Type 1	422,567	39,055	(307,959, 537,174)	10.82	0.000
Acct Type 3 - Acct Type 1	343,700	55,233	(181,620, 505,780)	6.22	0.000
Acct Type 4 - Acct Type 1	9492	49,144	(−134,720, 153,703)	0.19	1.000
Acct Type 5 - Acct Type 1	14,938	51,841	(−137,188, 167,064)	0.29	1.000
Acct Type 6 - Acct Type 1	11,367	65,740	(−181,547, 204,280)	0.17	1.000
Acct Type 3 - Acct Type 2	−78,867	59,658	(−253,933, 96,199)	−1.32	0.772
Acct Type 4 - Acct Type 2	−413,075	54,070	(−571,742, − 254,408)	−7.64	0.000
Acct Type 5 - Acct Type 2	−407,629	56,532	(−573,522, − 241,735)	−7.21	0.000
Acct Type 6 - Acct Type 2	−411,200	69,499	(−615,146, − 207,254)	−5.92	0.000
Acct Type 4 - Acct Type 3	−334,208	66,700	(−529,938, − 138,479)	−5.01	0.000
Acct Type 5 - Acct Type 3	−328,762	68,711	(−530,394, − 127,129)	−4.78	0.000
Acct Type 6 - Acct Type 3	−332,333	79,721	(−566,275, − 98,392)	−4.17	0.001
Acct Type 5 - Acct Type 4	5446	63,919	(−182,124, 193,017)	0.09	1.000
Acct Type 6 - Acct Type 4	1875	75,630	(−220,062, 223,812)	0.02	1.000
Acct Type 6 - Acct Type 5	−3571	77,410	(−230,731, 223,588)	− 0.05	1.000

The pairs with *p*-value that is less than 0.05 are significantly different from each other. For us to have a clearer picture, the result is summarized in Table 6.

**Table 6:** Grouping by Turkey's method.

Account Type	*N*	Mean	Grouping
Account Type 2 (Current)	15	444,200	A
Account Type 3 (Corporate)	6	365,333	A
Account Type 5	7	36,571	B
Account Type 6	4	33,000	B
Account Type 4	8	31,125	B
Account Type 1 (Savings)	30	216,33	B

**Remark**: The means that do not share the same letter are significantly different from each other

**Fig. 10:**
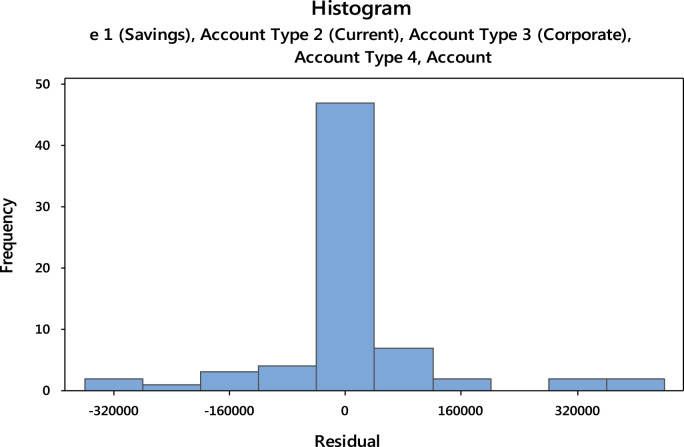
Plot for the residuals.

The residuals are represented in form of histogram and are displayed in Fig. 10.

**Fig. 11:**
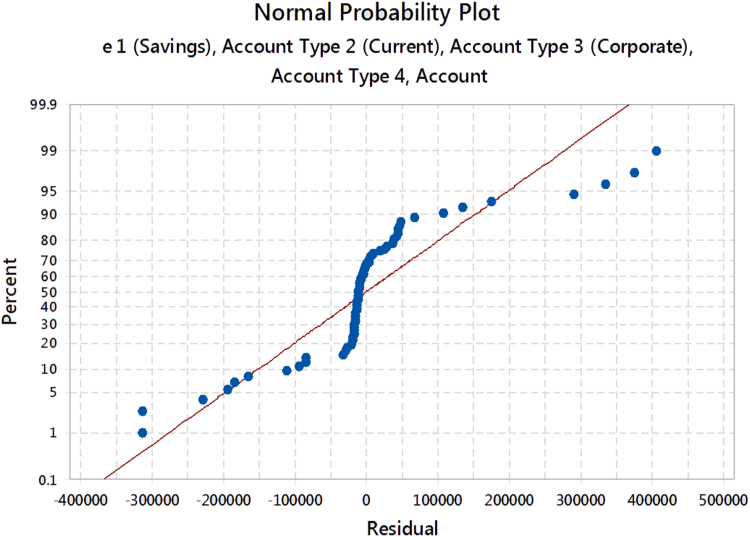
The normal probability plot for the residuals.

The normal probability plot for the residuals is displayed in Fig. 11.

## 3 Key information from the results

• The mean effect of current account and corporate account on the bank are the same.
• The mean effect of Savings account, account types 4, 5 and 6 on the bank are the same.
• Current account and corporate account attract more deposits than the other account types.

ANOVA has been applied to different research works which yielded some interesting results similar to this research [21], [22], [23], [24], [25].
